# The Role of Simultaneous Integrated Boost in Locally Advanced Rectal Cancer Patients with Positive Lateral Pelvic Lymph Nodes

**DOI:** 10.3390/cancers14071643

**Published:** 2022-03-24

**Authors:** Elisa Meldolesi, Giuditta Chiloiro, Roberta Giannini, Roberta Menghi, Roberto Persiani, Barbara Corvari, Claudio Coco, Stefania Manfrida, Carlo Ratto, Viola De Luca, Luigi Sofo, Sara Reina, Antonio Crucitti, Valeria Masiello, Nicola Dinapoli, Vincenzo Valentini, Maria Antonietta Gambacorta

**Affiliations:** 1 Department of Radiology, Radiation Oncology and Hematology, Catholic University of the Sacred Heart, Agostino Gemelli University Hospital Foundation IRCCS, 00168 Roma, Italy; elisa.meldolesi@policlinicogemelli.it (E.M.); giuditta.chiloiro@policlinicogemelli.it (G.C.); barbara.corvari@policlinicogemelli.it (B.C.); stefania.manfrida@policlinicogemelli.it (S.M.); viola.deluca@guest.policlinicogemelli.it (V.D.L.); sara.reina@guest.policlinicogemelli.it (S.R.); valeria.masiello@guest.policlinicogemelli.it (V.M.); nicola.dinapoli@policlinicogemelli.it (N.D.); vincenzo.valentini@policlinicogemelli.it (V.V.); mariaantonietta.gambacorta@policlinicogemelli.it (M.A.G.); 2 Digestive Surgery Unit, Catholic University of the Sacred Heart, Agostino Gemelli University Hospital Foundation IRCCS, 00168 Rome, Italy; roberta.menghi@policlinicogemelli.it (R.M.); roberto.persiani@policlinicogemelli.it (R.P.); claudio.coco@policlinicogemelli.it (C.C.); carlo.ratto@policlinicogemelli.it (C.R.); luigi.sofo@policlinicogemelli.it (L.S.); antonio.crucitti@policlinicogemelli.it (A.C.)

**Keywords:** locally advanced rectal cancer, lateral pelvic positive nodes, radiotherapy, simultaneous integrated boost, chemoradiotherapy

## Abstract

**Simple Summary:**

Around 15% of locally advanced rectal cancer patients have positive lateral pelvic lymph-nodes at diagnosis, with a worse prognosis due to the high incidence of distant metastasis. The best treatment modality for these patients is still a challenge. The aim of our retrospective study was to analyze the efficacy of the Simultaneous Integrated Boost—Intensity Modulated Radiation Therapy technique and determine the optimal dose of radiotherapy on clinically positive lateral pelvic lymph-nodes in locally advanced rectal cancer patients. Excellent results in terms of all the analyzed oncological outcomes have been observed. These results, if validated by future prospective studies, can bring a valid alternative to the surgery dissection without the important side effects and permanent disabilities observed during the years.

**Abstract:**

Aims: Between 11 to 14% of patients with locally advanced rectal cancer (LARC) have positive lateral pelvic lymph nodes (LPLN) at diagnosis, related to a worse prognosis with a 5-year survival rate between 30 to 40%. The best treatment choice for this group of patients is still a challenge. The optimal radiotherapy (RT) dose for LPLN patients has been investigated. Methods: We retrospectively collected data from LARC patients with LPLN at the primary staging MRI, treated in our center from March 2003 to December 2020. Patients underwent a neoadjuvant concomitant chemo-radiotherapy (CRT) treatment on the primary tumor (T), mesorectum, and pelvic nodes, associated with a fluoride-based chemotherapy. The total reached dose was 45 Gy at 1.8 Gy/fr on the elective sites and 55 Gy at 2.2 Gy/fr on the disease and mesorectum. Patients were divided in two groups based on whether they received a simultaneous integrated RT boost on the LPLN or not. Overall Survival (OS), Disease Free Survival (DFS), Metastasis Free Survival (MFS), and Local Control (LC) were evaluated in the whole group and then compared between the two groups. Results: A total of 176 patients were evaluated: 82 were included in the RT boost group and 94 in the non-RT boost group. The median follow-up period was 57.8 months. All the clinical endpoint (OS, DFS, MFS, LC), resulted were affected by the simultaneous integrated boost on LPLN with a survival rate of 84.7%, 79.5%, 84.1%, and 92%, respectively, in the entire population. From the comparison of the two groups, there was a statistical significance towards the RT boost group with a *p* < 0.006, 0.030, 0.042, 0.026, respectively. Conclusions: Concomitant radiotherapy boost on positive LPLN has shown to be beneficial on the survival outcomes (OS, DFS, MFR, and LC) in patients with LARC and LPLN. This analysis demonstrates that a higher dose of radiotherapy on positive pelvic lymph nodes led not only to a higher local control but also to a better survival rate. These results, if validated by future prospective studies, can bring a valid alternative to the surgery dissection without the important side effects and permanent disabilities observed during the years.

## 1. Introduction

The management of locally advanced rectal cancer (LARC) has progressively evolved in recent decades with the increasing use of neoadjuvant chemoradiotherapy (nCRT) [1,2]. Currently, the gold standard for the treatment of locally advanced rectal cancer is radiotherapy with or without chemotherapy followed by radical surgical resection as anterior rectal resection (RA) or abdominal-perineal excision (APR), according to the principle of total mesorectal excision (TME) [3]. Although this approach significantly reduced the local recurrence rate compared to exclusive surgery [4], some patients still developed lateral pelvic recurrence [5,6,7]. Involvement of extramesorectal lymph nodes occurs in 7–15% of cases of locally advanced rectal cancer. It is particularly common in cases of cT3-4 with positive nodes in the mesorectum or distal rectal disease [8,9,10]. The optimal treatment modality for positive extramesorectal lymph nodes still remains unclear. Surgical dissection of the lateral pelvic lymph nodes (LPLD) is recommended in some guidelines, however LPLD is associated with long surgical times and may increase the risk of urinary and sexual function related undesirable effects [11,12,13]. Kusters, M et al. compared the possibility of local relapse in patients with LARC between neoadjuvant chemoradiotherapy (NCRT) and LPLD, resulting in similar relapse rates [14]. LPLD could be an overtreatment for LPLN negative patients.

Although MRI is considered the diagnostic technique with the greatest diagnostic sensitivity on determining the positivity of the lymph nodes at the level of the pelvic excavation, there is still no international consensus on the criteria to be followed to identify metastatic lymph nodes. Ogawa et al. reported that a short axis >5 mm of the lymph nodes has a predictive power of positivity with an accuracy of about 80% [15]. Akiyoshi et al. demonstrated that LPLNs with a short axis diameter of ≥8 mm is associated with a higher metastasis rate, even after NCRT [16]. Atsushi et al. indicated that a short axis of 7 mm could be a risk factor for local lateral recurrence [7]. More recently, the European guidelines of the Abdominal and Gastrointestinal Radiological Society (ESGAR) have highlighted how, alongside dimensional criteria, it is also fundamental to take morphological criteria into account, increasing the accuracy of predicting lymph node positivity [17]. Taken together, however, these data suggest that standard nCRT may be insufficient treatment in the presence of positive LPLNs.

Radiotherapy dose escalation could be a non-surgical strategy to improve local treatment outcomes for positive LPLN. The simultaneous integrated boost (SIB) technique, which involves the simultaneous administration of different doses to different target areas, has been widely used in lung cancer and some abdominal cancers [18,19,20].

This study is aimed at analyzing the efficacy of the SIB-Intensity Modulated Radiation Therapy (IMRT) technique and determining the optimal dose of radiotherapy on clinically positive LPLNs in LARC patients.

## 2. Materials and Methods

### 2.1. Patients

176 patients (105 men, 71 females, with an age over 50 years at the diagnosis in 89.8% of the cases) with locally advanced non-metastatic rectal cancer were enrolled in this observational, retrospective, and monocentric study. Inclusion criteria are reported in Table 1. All patients underwent neoadjuvant chemoradiotherapy for low-medium-upper LARC at the Department of Oncological Radiotherapy—Gemelli Art at the University Hospital A. Gemelli IRCCS in Rome between March 2003 to January 2020. All cases were discussed in the multidisciplinary meeting of rectal pathology, attended by radiation oncologists, medical oncologists, radiologists, pathologists, and surgeons, to identify the best therapeutic options, both at the time of the diagnosis and at the re-evaluation after 6–8 weeks from the neoadjuvant therapy. The 7th edition of the TNM was used for staging and shared restaging. One-year follow-up was mandatory to be included in the study.

### 2.2. Imaging

**Figure 1:**
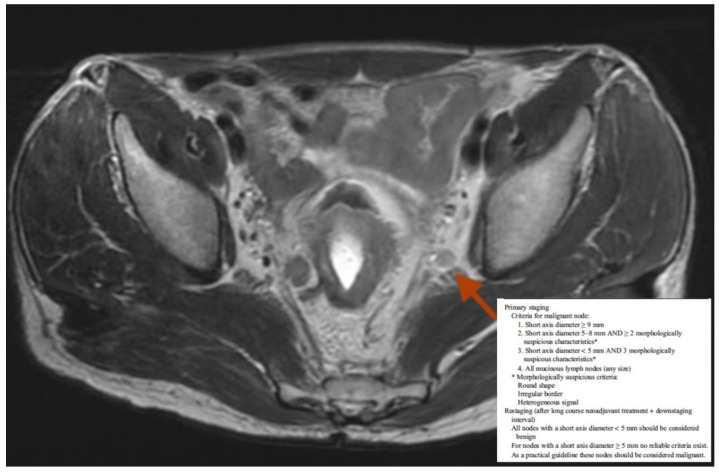
Practical guidelines for nodal staging (ESGAR guidelines).

At the primary staging, all patients underwent both pelvic MRI and chest and abdominal CT, while FDG-PETCT was considered and performed only in a few selected cases. Dimensional, morphological, and signal criteria were used to identify positive lateral pelvic lymph node at the MRI according to the practical guidelines recommended by the European Society of Gastrointestinal and Abdominal Radiology [17] Figure 1. After 6–8 weeks from the end of the nCRT, all patients were re-evaluated with a pelvic MRI for local restaging and a chest and abdominal CT in high-risk patients (extramesorectal lymph nodes, mucinous tumor, and EMVI or ExtraMural Vascular Invasion).

### 2.3. Neoadjuvant Treatment

All patients underwent neoadjuvant chemo-radiotherapy treatment with either long or short course radiotherapy. Chemotherapy based on fluoropyrimidine ± oxaliplatin, depending on the stage of the disease, was given.

### 2.4. Surgery

Surgical treatment was performed including a Total Mesorectal Excision (TME)/Partial Mesorectal Excision (PME) technique, according to the initial location of the tumor (low, mid, high) and/or the amount of the disease after the restaging. Rectal resection with TME was performed according to the standard technique using the open or laparoscopic approach and included both anterior resection of the rectum and resection of the rectum via the abdominoperineal route. In rare cases, a Hartmann’s procedure was executed. In case of local excision, it could be performed with traditional techniques (TAE, TEM, TAMIS, etc.).

#### Clinical Workflow

**Figure 2:**
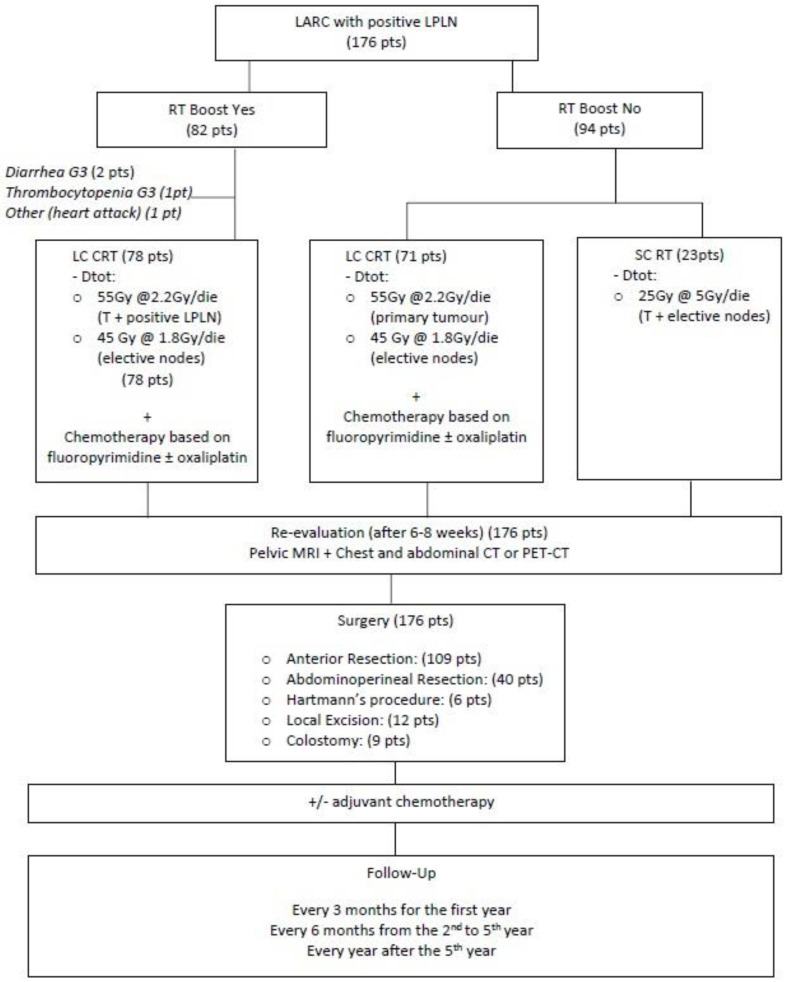
Flowchart of the clinical workflow. LARC: Locally Advanced Rectal Cancer; LPLN: Lateral Pelvic Lymph Nodes; RT boost yes/no: Radiotherapy boost yes/no; LC CRT: Long Course chemoradiotherapy; SC CRT: Short Course chemoradiotherapy.

A flowchart of the clinical workflow is represented in Figure 2.

Patients were divided in two groups based on whether they received a simultaneous integrated RT boost on the LPLN or not.

**Figure 3:**
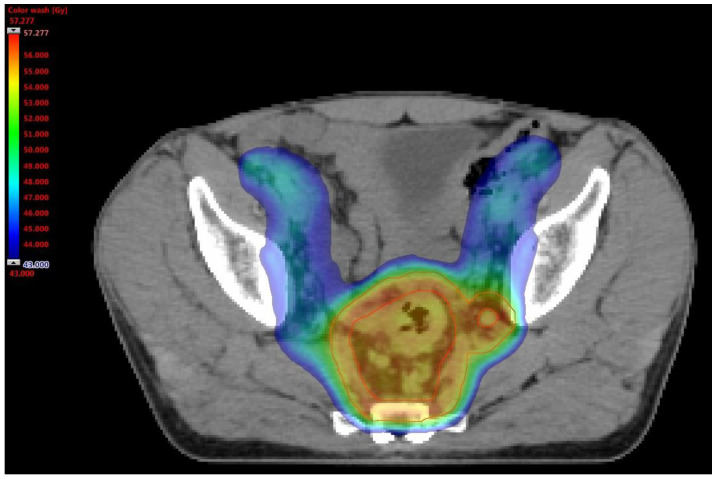
Dose distribution in a LCRT with positive LPLN.

The first group underwent a long course RT schedule. A total dose of 45 Gy at 1.8 Gy/day was given to the elective sites and a total dose of 55 Gy at 2.2 Gy/day was reached on the disease, corresponding mesorectum and clinically positive LPLNs with a SIB technique. (Figure 3).

Besides, the group who did not received the RT boost underwent either long or short radiotherapy schedule. A total dose of 45 Gy at 1.8 Gy/day on the elective sites with 55 Gy at 2.2 Gy/day on the disease and corresponding mesorectum was delivered for the long course, whereas a total dose of 25 Gy at 5 Gy/day on the disease, whole mesorectum, and elective sites was reached in the short course.

The radiation treatment was delivered by means of either three-dimensional conformational radiotherapy techniques (3D-CRT) or IMRT according to the time they were treated (before or after 2010, respectively).

The concomitant chemotherapy treatment, on the other hand, was carried out with the administration of difluoropyrimidines according to different therapeutic schemes:

- oxaliplatin iv 50 mg/m^2^/day 1, 8, 21, 28, and 5-Fluorouracil 250 mg/m^2^/day days 1–7 during the 1st–2nd and 4th–5th week of radiotherapy
- oxaliplatin iv 60 mg/m^2^/day g1 q7 and capecitabine per os 1300 mg/m^2^/day 1–7 days
- 5-fluorouracil iv 225 mg/m^2^/day in continuous infusion days 1–7 q7
- capecitabine per os 1650 mg/m^2^/day 1–7 q7
- capecitabine per os 1650 mg/m^2^/day days 1–5 q7 and avelumab iv 10 mg/kg/dieg1 q14

Clinical response was assessed with pelvic MRI and chest and abdominal CT or FDG-PETCT, performed between 6 to 8 weeks after the end of nCRT.

During the last decade, in case of mCR or cCR at instrumental re-evaluation, a second evaluation with MRI and endoscopy could be performed between 12 and 14 weeks after the end of nCRT.

### 2.5. Follow-Up

As follow-up, patients were evaluated every 3 months for the first year, every 6 months from the 2nd to the 5th years, and every year after the 5th year.

### 2.6. Statistical Analysis

All the variables under study will be summarized using descriptive statistical techniques. In particular, the qualitative variables will be summarized through absolute and percentage frequencies. There distribution of quantitative variables will be verified through the Shapiro–Wilk test.

The differences between the two groups at the baseline will be evaluated, as regards the variables qualitative, through Fisher’s exact test or Chi Square test, as appropriate. The variables quantitative will instead be evaluated through the Student’s *t* test, in case of data normally distributed, or via Mann–Whitney’s U-test, otherwise.

For survival outcomes, the analyzes will concern the assessment of time to events death, systemic recurrence of disease, and local recurrence. The survival outcome is defined as the time elapsed from the date of surgery to the date of the event. In the absence of the event, it will be considering the date of the last visit. If the date of the surgery is not available, it will be replaced by the date of the revaluation MRI. Considered outcomes are death, local recurrence (recurrence of disease in the pelvis: intraluminal or extraluminal), and distant recurrence (disease recurrence in any other location). The diagnosis of relapse will be determined based on the clinical examination, radiological images, or biopsy. Survival will be estimated by analysis of survival through the Kaplan–Meier method and the Log-rank will be used for the comparison between the two groups. The results will be expressed with relative 95% confidence intervals.

A *p*-value < 0.05 will be considered statistically significant. All analyses will be conducted through STATA version 16 software (STATA Corp, College Station, TX, USA). The study was an Internal Review Board (IRB) approved study (registrar number: 0045273/21 of 23 December 2021)

## 3. Results

From March 2003 to December 2020, a total of 176 patients with locally advanced non-metastatic rectal cancer were evaluated: 82 (46.6%) were included in the RT boost group and 94 (53.4%) in the non-RT boost group.

The median follow-up period was 57.8 months (range 52.3–63.1).

Patients’ characteristics in the two groups are reported in Table 2.

No differences were observed between the two groups, except for vascular invasion (EMVI) and mucinous histotype. This illusory result is actually related to the fact that the study is a retrospective study where most of the patients who did not undergo to a RT boost were treated before 2010 when some data as EMVI and mucinous characteristics were not recorded. This is the reason for a higher number of non-available data in the no RT boost group.

Seventy-eight patients (95.1%) from the RT boost group reached 55 Gy on T and LPLN, 4 patients (4.9%) had to stop CTRT earlier due to grade 3 GI toxicity in 1 case and grade 3 thrombocytopenia in 1 case, and one patient died unexpectedly during the RTCT due to heart attack. Total delivered dose on elective pelvic nodes was 45 Gy in all RT boost group patients.

Twenty-three patients (24.2%) in the no RT boost group received a total dose on T < 55 Gy: 12 patients underwent a short course schedule 25 Gy @ 5 Gy/die; from the remaining 11 patients, 9 patients were prescribed as a total dose of 50.4 Gy, 1 patient of 45 Gy and 1 patients initially prescribed as short course, had to stop nCRT at 20 Gy due to grade 3 GI toxicity. Total delivered dose on elective pelvic nodes and LPLN in the no RT boost was 45 Gy in 82 patients (86.3%); 25 Gy in 12 patients (12.6%) treated with the short course schedule and 20 Gy in 1 patient (1.1%) initially prescribed as short course but interrupted beforehand due to grade 3 GI toxicity.

Rectal resection with TME was performed using an anterior resection (AR) procedure in 53 patients (64.6%) and in 56 patients (59.6%) in the RT boost and in the no RT boost group, respectively. Twenty patients in both groups (24.4% and 21.3%) underwent a resection of the rectum via the abdominoperineal route. In rare cases, a Hartmann’s procedure was executed: 1 case (1.2%) in the RT boost group and 5 cases (5.3%) in the no RT boost group. Local excision was performed in 7 patients (8.5%) in the RT boost and in 5 patients (5.3%) in the no RT boost group, in case of clinical major or complete response of a tumor located in the in lower 1/3 of the rectum. Finally, 9 patients underwent only a colostomy surgery due to a clinical and endoscopical complete response in 1 patient (11.1%) from the no RT boost group, due to a progression disease in 6 patients (66.7%)—5 patients in the no RT boost and 1 patient in the RT boost group, and due to high risk for comorbidities in 2 patients (22.2%) in the no RT boost group.

Twenty-seven patients (15.3%) died at the follow-up: 23 (85.2%) in the no RT boost and 4 (14.8%) in the RT boost group (*p* = 0.006).

Twenty-eight patients (15.9%) developed distant metastasis at some point: 21 patients (75%) in the no RT boost group and 7 patients (25%) in the RT boost group (*p* = 0.031). Thirty-six patients (20.5%) had a local (either local or regional) and/or distant relapse: 28 (77.8%) in the no RT boost and 8 (22.2%) in the RT boost group (*p* = 0.004).

Finally, 14 patients (7.9%) had a local (either local or regional) relapse: 12 patients (85.7%) in the no RT boost group and 2 patients (14.3%) in the RT boost group, respectively (*p* = 0.026).

**Figure 4:**
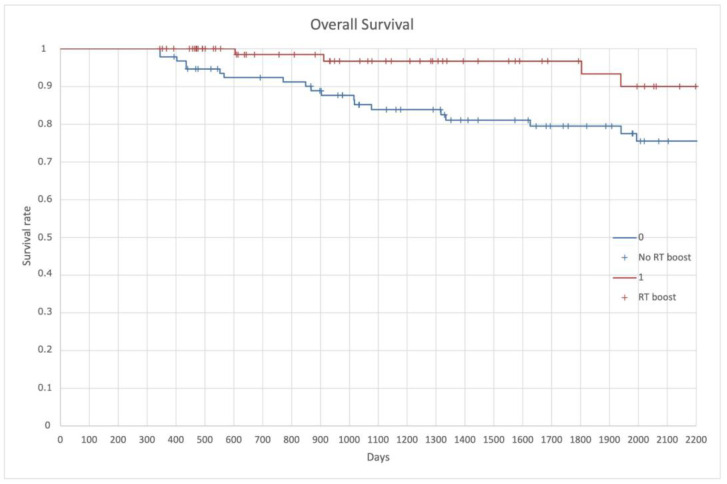
Overall Survival (OS).

All the clinical endpoint resulted affected by the simultaneous integrated boost on LPLN. OS resulted in 84.7% in the entire population with a 3-year and 5-year of 85% and 75%, respectively, in the no RT boost, and 96% and 90%, respectively, in the RT boost group (*p* = 0.006) (Figure 4).

**Figure 5:**
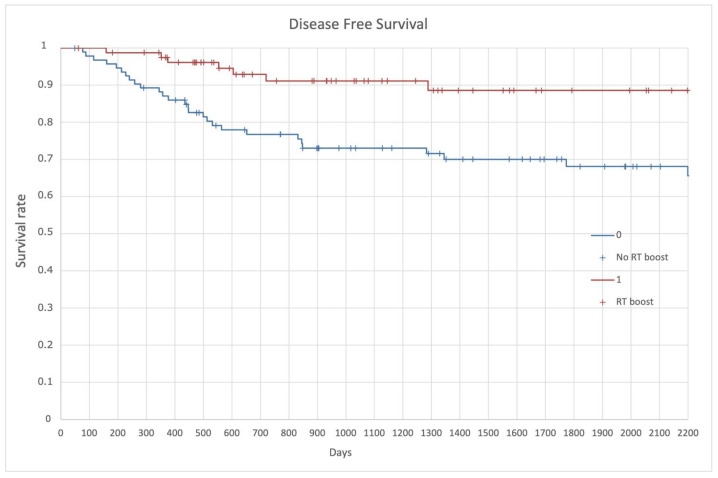
Disease Free Survival (DFS).

DFS resulted in 79.5% in the whole population, with a 3-year and 5-year of 73% and 68%, respectively, in the no RT boost, and 91% and 88%, respectively, in the RT boost group (*p* = 0.03) (Figure 5).

**Figure 6:**
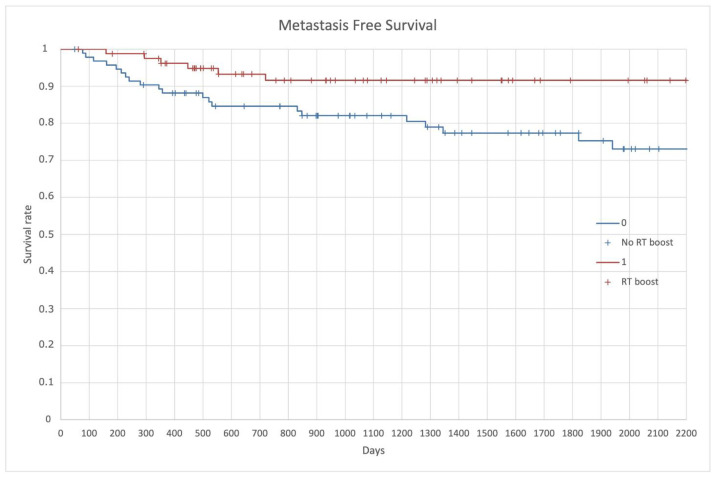
Metastasis Free Survival (MFS).

Distant metastasis appeared in 15.9% of the entire population; patients in the no RT group had higher risk to develop distant metastasis (22.3%) than in the RT boost group (8.5%) (*p* = 0.04). MFS at 3-years and 5-years were 82% and 75% in the no RT boost, while it was constantly on 91% in the RT boost group at 3- and 5-years (*p* = 0.04) (Figure 6).

**Figure 7:**
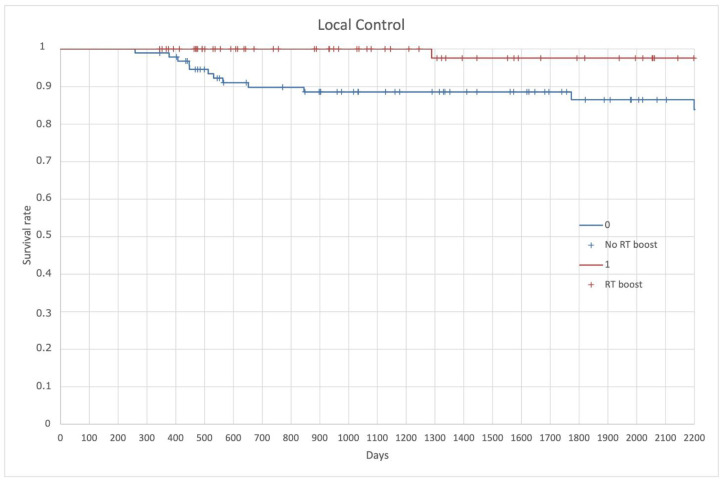
Local control (LC).

Finally, LC resulted in 92% in the entire population. LC at 3-year and 5-year was 88% and 86%, respectively, for the no RT boost and 99% and 97%, respectively, for the RT boost group (*p* = 0.026) (Figure 7).

Univariate and multivariate analysis results are depicted in Table 3.

Total delivered dose on LPLN was the only feature that resulted as statistically significant on all the clinical outcomes of OS, DFS, MFS, and LC (*p* < 0.01; <0.01; 0.04; 0.04), along with cT which resulted as statistically significant on OS, DFS, and MFS (*p* = 0.04, 0.03, and 0.04, respectively). Surgery type has resulted affecting both DFS and LC. Finally, at the multivariate analysis, both total delivered dose on LPLN and cT were confirmed as important features, affecting all the clinical outcomes except for LC, where they did not reach the statistical significance (*p* = 0.08, 0.16). Surgery type maintained its statistical significance on LC. cT4 had a higher risk to develop metastasis (*p* = 0.01) leading to lower DFS and OS (*p* < 0.01, 0.01). A simultaneous integrated boost with a total delivered dose on LPLN equal to 55 Gy gave a significant gain in terms of higher OS, DFS, and MFS (*p* = 0.03, 0.01, 0.04).

## 4. Discussion

The best treatment modality for LARC patients with positive LPLN at the diagnosis is still a challenge because of the high incidence of distant metastasis and poor survival. There is currently a lack of randomized data to guide how lateral pelvic lymph nodes should be managed in patients with rectal cancer. The available observational data are of limited quality, but suggest that sterilization of all lateral pelvic nodes reduces the risk of local recurrence [21]. However, there is still the question of how such sterilization is reached. The role of prophylactic lateral lymph nodes dissection (LLND) compared to neoadjuvant chemoradiation (nCRT) is still a topic of ongoing debate [22,23]. Neoadjuvant chemoradiation therapy (CRT) followed by total mesorectal excision (TME) is the current standard of care for LARC in North America and Europe [22], even though the long-term advantages of this approach have not been fully established yet [24,25]. LLND, first used in Western countries in the 1950s and then abandoned because of its significant morbidity and postoperative functional disabilities [26], is still part of the standard of care of LARC patients with LPLN in eastern countries, such as Japan, where Japanese guidelines add it to the routinely used surgical therapy [27]. Furthermore, controversies are still open on how to reliably detect positive lateral pelvic lymph node. MRI has been shown to be superior to clinical examination, computer tomography, and endoluminal ultrasound (EUS) for rectal tumor staging [28], and to better characterize suspicious lymph nodes [29]. MRI is considered highly accurate in detecting lateral pelvic nodes, with a 67% sensitivity, 75% specificity, and 73% overall accuracy [21]. Some groups such as LOREC (Low Rectal Cancer Study Group), or the Japanese Society for Cancer of the Colon and Rectum, suggest nodal size as the main feature in order to determine which nodes might be considered as pathological with different cutoffs (>5, >7, >8 mm on the long or short axis) [15,30]. However, to date no solid evidence regarding specific or alternative (size) criteria for extramesorectal nodes are reported and it is not feasible to recommend any specific criteria for these nodes. In this study, both dimensional and morphological criteria were used to identify positive lateral pelvic lymph nodes on MRI, according to the practical guidelines recommended by the European Society of Gastrointestinal and Abdominal Radiology [17].

During the last decades, going from a standard 3-dimensional (3D) conformal radiation therapy to more highly conformal treatment approaches such as an Intensity Modulated Radiation Therapy (IMRT) and Volumetric Modulated Arc Therapy (VMAT) has led to a superior homogeneity and conformity of dose distribution in target volumes with better sparing of the organ at risk [31,32]. Furthermore, encouraging results in terms of pathologic complete response (pCR) and local control (LC) with dose escalation using a simultaneous integrated boost (SIB) technique have been published [33]. Because of this promising impact on clinical outcomes but conflicting acute and late toxicity’s results, mainly related both to the addition of oxaliplatin to the standard neoadiuvant concomitant capecitabine and radiotherapy dose escalation, But-Hadzic et al. tested this hypofractionated technique in order to shorten the overall treatment time with a biologically effective dose (BED) similar to the standard 3D CRT one [34]. The result was a high pCR rate with a very favorable acute toxicity profile.

Recent studies testing the effect of the SIB-IMRT in LARC patients with clinically positive lateral pelvic lymph-nodes [35,36] have demonstrated that a SIB on LPLN could be an effective strategy to eliminate clinical positive lateral pelvic nodes without increasing the radiotherapy-related toxicity. Li at al. retrospectively evaluated the role of the SIB technique in terms of regrowth rate and radiation-related toxicity in a population of 151 patients. They found that SIB-IMRT chemo-radiotherapy is beneficial for eliminating clinical positive LPLN from LARC without increasing the incidence of RT-related toxicity and surgical complications, especially for larger pelvic nodes.

To the best of our knowledge, the present study is the first to evaluate the role of a simultaneous integrated RT boost on LPLN on oncological outcomes (OS; DFS; MFS; LC).

In our institution, SIB-IMRT technique is used as a standard nCRT technique in rectal cancer to obtain a higher dose at the site of the disease and a lower dose for microscopic disease control. Since 2003, some LARC patients with positive LPLN have also undergone a SIB-IMRT on LPLNs to increase the local response to treatment on those lymph nodes that would not have been surgically removed at the time of surgery on the primary rectal.

In this analysis, we found that escalating the dose to 55 Gy on positive LPLN using SIB-IMRT appeared to be beneficial in terms of all the considered oncological outcomes.

Some objections could be made as to the appropriateness of including patients who underwent short course radiotherapy in the no RT boost group, due to the possibility of that influencing the outcomes. Considering that the aim of the study was to evaluate if a higher dose on lateral positive lymph nodes could provide better oncological outcomes than standard dose and considering that previous phase III randomized studies have demonstrated the comparable efficacy of a long course versus a short course treatment in terms of oncological outcomes [37,38], we decided it was appropriate to also include SCRT patients. Intrinsic limitation of this study is mainly related to the fact that is a retrospective monocentric study that includes a wide heterogeneity of patients and treatments over the years. Moreover, the impossibility of having part of the EMVI and mucinous information, mainly for the no RT boost patients, could be responsible for a possible impact on imbalance for the higher incidence of metastasis or DFS and possible OS of this group. Furthermore, late toxicity analysis and chemotherapy evaluation was not possible due to a lack of data. A more comprehensive analysis of the chemotherapy treatments could help in comprehending the obtained lower incidence rate of metastasis with respect to the literature. The difficult role of MRI in the identification of positive pelvic lymph node at the diagnosis could also be responsible for this result. In fact, although MRI is still considered to be the diagnostic technique with the greatest diagnostic sensitivity for determining the positivity of the lymph nodes at the level of the pelvic excavation, there is still no international consensus on the criteria to be followed to identify metastatic lymph nodes.

Although these are limitations, this analysis demonstrates that a higher dose of radiotherapy on positive pelvic lymph nodes can lead not only to a higher local control but also to a better survival rate. These results, if validated by future prospective studies, can bring a valid alternative to the surgical dissection without the important side effects and permanent disabilities observed during the years.

## 5. Conclusions

In conclusion, excellent results in terms of OS, DFS, MFS, and LC after preoperative treatment of LARC with clinically positive LPLN with IMRT-SIB in 25 fractions have been observed. These results, if validated by future prospective studies, can bring a valid alternative to the surgery dissection without the important side effects and permanent disabilities observed during the years.

## Figures and Tables

**Table 1:** Inclusion criteria.

**Inclusion Criteria**
• Histologically documented adenocarcinoma of the rectum; • Minimum age of 18; • Locally advanced non-metastatic rectal cancer; • Undergoing neoadjuvant CRT treatment followed or not by TME surgery; • MRI pelvis staging and restaging available; • Follow-up of at least one year; • Signature of informed consent to the processing of their data, if applicable.
**Exclusion Criteria**
• Patients treated for palliative purposes; • Patients with metastatic disease.

**Table 2:** Patients’ characteristics divided by RT boost’s groups.

Patient’s Characteristics	Boost Yes	Boost No	*p* Value ^§^
**Sex**			0.368
Male	46 (56.1%)	59 (62.8%)	
Female	36 (43.9%)	35 (37.2%)	
**Age**			0.760
≤50 years	9 (11%)	9 (9.6%)	
<50 years	73 (89%)	85 (90.4%)	
**cT**			0.644
2	1 (1.2%)	2 (2.1%)	
3	49 (59.8%)	50 (53.2%)	
4	32 (39%)	42 (44.7%)	
**cN**			0.577
1	19 (23.2%)	21 (22.3%)	
2	63 (76.8%)	73 (77.7%)	
**EMVI**			0.001
+	9 (11%)	5 (5.3%)	
−	71 (86.6%)	69 (73.4%)	
N/A	2 (2.4%)	20 (21.3%)	
**Mucinous**			0.003
Yes	4 (4.9%)	6 (6.4%)	
No	76 (92.7%)	71 (75.5%)	
N/A	2 (2.4%)	17 (18.1%)	
**MRF**			0.834
+	40 (48.8%)	48 (51.1%)	
−	38 (46.3%)	43 (45.7%)	
N/A	4 (4.9%)	3 (3.2%)	
**Surgery Type**			0.101
Anterior Resection (AR)	53 (64.6%)	56 (59.6%)	
Abdominoperineal Resection (APR)	20 (24.4%)	20 (21.3%)	
Hartmann	1 (1.2%)	5 (5.3%)	
Local Excision	7 (8.5%)	5 (5.3%)	
Colostomy	1 (1.2%)	8 (8.5%)	
**RT dose on T**			<0.001
55 Gy	78 (95.1%)	71 (75.5%)	
<55 Gy	4 (4.9%)	23 (24.5%)	
**RT dose on LPLN**			8.45
55 Gy	78 (95.1%)	0 (0%)	
<55 Gy	4 (4.9%)	94 (100%)	

^§^ χ^2^ test.

**Table 3:** Univariate and multivariate analysis.

Patient’s Characteristics	OS		DFS		MFS		LC	
	Univariate	Multivariate	Univariate	Multivariate	Univariate	Multivariate	Univariate	Multivariate
	*p*-Value	*p*-Value	*p*-Value	*p*-Value	*p*-Value	*p*-Value	*p*-Value	*p*-Value
Gender	0.09		0.29		0.42		0.44	
Age	0.50		0.25		0.24		0.27	
cT	0.04	0.01	0.03	0.05	0.04	0.02	0.22	0.16
cN	0.74		0.37		0.07		0.58	
EMVI	0.46		0.98		0.99		0.99	
Mucinous	0.63		0.98		0.99		0.99	
Mesorectal Fascia (MRF)	0.63		0.61		0.37		0.15	
Surgery Type	0.17		0.02	0.07	0.29		<0.001	0.01
RT dose on T	0.04	0.06	0.49		0.77		0.15	
RT dose on LPLN	<0.01	0.03	<0.01	0.01	0.04	0.04	0.04	0.08

## Data Availability

The data presented in this study are available on request from the corresponding author.

## References

[1] Aly E. (2014). Time for a Renewed Strategy in the Management of Rectal Cancer: Critical Reflection on the Surgical Management of Rectal Cancer over 100 Years. Dis. Colon Rectum.

[2] On J., Aly E.H. (2018). “Watch and Wait” in Rectal Cancer: Summary of the Current Evidence. Int. J. Colorectal Dis..

[3] Nelson H., Petrelli N., Carlin A., Couture J., Fleshman J., Guillem J., Miedema B., Ota D., Sargent D. (2001). Guidelines 2000 for Colon and Rectal Cancer Surgery. J. Natl. Cancer Inst..

[4] Koukourakis G.V. (2012). Role of Radiation Therapy in Neoadjuvant Era in Patients with Locally Advanced Rectal Cancer. World J. Gastrointest. Oncol..

[5] Shiratori H., Kawai K., Hata K., Tanaka T., Nishikawa T., Sasaki K., Kaneko M., Murono K., Emoto S., Morikawa T. (2019). Correlations between the Recurrence Patterns and Sizes of Lateral Pelvic Lymph Nodes before and after Chemoradiotherapy in Patients with Lower Rectal Cancer. Oncology.

[6] Kim M.J., Kim T.H., Kim D.Y., Kim S.Y., Baek J.Y., Chang H.J., Park S.C., Park J.W., Oh J.H. (2015). Can Chemoradiation Allow for Omission of Lateral Pelvic Node Dissection for Locally Advanced Rectal Cancer?. J. Surg. Oncol..

[7] Ogura A., Konishi T., Cunningham C., Garcia-Aguilar J., Iversen H., Toda S., Lee I.K., Lee H.X., Uehara K., Lee P. (2019). Neoadjuvant (Chemo)Radiotherapy With Total Mesorectal Excision Only Is Not Sufficient to Prevent Lateral Local Recurrence in Enlarged Nodes: Results of the Multicenter Lateral Node Study of Patients With Low CT3/4 Rectal Cancer. J. Clin. Oncol..

[8] Hida J., Yasutomi M., Fujimoto K., Maruyama T., Okuno K., Shindo K. (1997). Does Lateral Lymph Node Dissection Improve Survival in Rectal Carcinoma? Examination of Node Metastases by the Clearing Method. J. Am. Coll. Surg..

[9] Yagi R., Shimada Y., Kameyama H., Tajima Y., Okamura T., Sakata J., Kobayashi T., Kosugi S., Wakai T., Nogami H. (2016). Clinical Significance of Extramural Tumor Deposits in the Lateral Pelvic Lymph Node Area in Low Rectal Cancer: A Retrospective Study at Two Institutions. Ann. Surg. Oncol..

[10] Sugihara K., Kobayashi H., Kato T., Mori T., Mochizuki H., Kameoka S., Shirouzu K., Muto T. (2006). Indication and Benefit of Pelvic Sidewall Dissection for Rectal Cancer. Dis. Colon Rectum.

[11] Fujita S., Akasu T., Mizusawa J., Saito N., Kinugasa Y., Kanemitsu Y., Ohue M., Fujii S., Shiozawa M., Yamaguchi T. (2012). Postoperative Morbidity and Mortality after Mesorectal Excision with and without Lateral Lymph Node Dissection for Clinical Stage II or Stage III Lower Rectal Cancer (JCOG0212): Results from a Multicentre, Randomised Controlled, Non-Inferiority Trial. Lancet Oncol..

[12] Georgiou P., Tan E., Gouvas N., Antoniou A., Brown G., Nicholls R.J., Tekkis P. (2009). Extended Lymphadenectomy versus Conventional Surgery for Rectal Cancer: A Meta-Analysis. Lancet Oncol..

[13] Saito S., Fujita S., Mizusawa J., Kanemitsu Y., Saito N., Kinugasa Y., Akazai Y., Ota M., Ohue M., Komori K. (2016). Male Sexual Dysfunction after Rectal Cancer Surgery: Results of a Randomized Trial Comparing Mesorectal Excision with and without Lateral Lymph Node Dissection for Patients with Lower Rectal Cancer: Japan Clinical Oncology Group Study JCOG0212. Eur. J. Surg. Oncol..

[14] Kusters M., Beets G., van de Velde C.J.H., Beets-Tan R.G.H., Marijnen C., Rutten H.J.T., Putter H., Moriya Y. (2009). A Comparison between the Treatment of Low Rectal Cancer in Japan and the Netherlands, Focusing on the Patterns of Local Recurrence. Ann. Surg..

[15] Ogawa S., Hida J.-I., Ike H., Kinugasa T., Ota M., Shinto E., Itabashi M., Kameoka S., Sugihara K. (2016). Selection of Lymph Node-Positive Cases Based on Perirectal and Lateral Pelvic Lymph Nodes Using Magnetic Resonance Imaging: Study of the Japanese Society for Cancer of the Colon and Rectum. Ann. Surg. Oncol..

[16] Akiyoshi T., Matsueda K., Hiratsuka M., Unno T., Nagata J., Nagasaki T., Konishi T., Fujimoto Y., Nagayama S., Fukunaga Y. (2015). Indications for Lateral Pelvic Lymph Node Dissection Based on Magnetic Resonance Imaging Before and After Preoperative Chemoradiotherapy in Patients with Advanced Low-Rectal Cancer. Ann. Surg. Oncol..

[17] Beets-Tan R.G.H., Lambregts D.M.J., Maas M., Bipat S., Barbaro B., Curvo-Semedo L., Fenlon H.M., Gollub M.J., Gourtsoyianni S., Halligan S. (2018). Magnetic Resonance Imaging for Clinical Management of Rectal Cancer: Updated Recommendations from the 2016 European Society of Gastrointestinal and Abdominal Radiology (ESGAR) Consensus Meeting. Eur. Radiol..

[18] Han D., Qin Q., Hao S., Huang W., Wei Y., Zhang Z., Wang Z., Li B. (2014). Feasibility and Efficacy of Simultaneous Integrated Boost Intensity-Modulated Radiation Therapy in Patients with Limited-Disease Small Cell Lung Cancer. Radiat. Oncol..

[19] Jeter M.D., Gomez D., Nguyen Q.N., Komaki R., Zhang X., Zhu X., O’Reilly M., Fossella F.V., Xu T., Wei X. (2018). Simultaneous Integrated Boost for Radiation Dose Escalation to the Gross Tumor Volume with Intensity-Modulated (Photon) Radiation Therapy or Intensity-Modulated Proton Therapy and Concurrent Chemotherapy for Stage II–III Non-Small Cell Lung Cancer: A Phase I Study. Int. J. Radiat. Oncol. Biol. Phys..

[20] Teng F., Meng L., Zhu F., Ren G. (2021). Dosimetric Feasibility on Hypofractionated Intensity-Modulated Radiotherapy and Simultaneous Integrated Boost for Locally Advanced Unresectable Pancreatic Cancer with Helical Tomotherapy. J. Gastrointest. Oncol..

[21] Matsuoka H., Nakamura A., Masaki T., Sugiyama M., Nitatori T., Ohkura Y., Sakamoto A., Atomi Y. (2007). Optimal Diagnostic Criteria for Lateral Pelvic Lymph Node Metastasis in Rectal Carcinoma. Anticancer Res..

[22] Benson A.B., Venook A.P., Al-Hawary M.M., Cederquist L., Chen Y.J., Ciombor K.K., Cohen S., Cooper H.S., Deming D., Engstrom P.F. (2018). Rectal Cancer, Version 2.2018 Clinical Practice Guidelines in Oncology. JNCCN J. Natl. Compr. Cancer Netw..

[23] Emile S.H., Elfeki H., Shalaby M., Sakr A., Kim N.K. (2021). Outcome of Lateral Pelvic Lymph Node Dissection with Total Mesorectal Excision in Treatment of Rectal Cancer: A Systematic Review and Meta-Analysis. Surgery.

[24] Santiago I., Pares O., Carvalho C., Figueiredo N., Heald R.J. (2017). The Perfect Total Mesorectal Excision Obviates the Need for Anything Else in the Management of Most Rectal Cancers. Clin. Colon Rectal Surg..

[25] Inoue Y., Saigusa S., Hiro J., Toiyama Y., Araki T., Tanaka K., Mohri Y., Kusunoki M. (2016). Clinical Significance of Enlarged Lateral Pelvic Lymph Nodes before and after Preoperative Chemoradiotherapy for Rectal Cancer. Mol. Clin. Oncol..

[26] Petersen S.H., Harling H., Kirkeby L.T., Wille-Jørgensen P., Mocellin S. (2012). Postoperative Adjuvant Chemotherapy in Rectal Cancer Operated for Cure. Cochrane Database Syst. Rev..

[27] Watanabe T., Muro K., Ajioka Y., Hashiguchi Y., Ito Y., Saito Y., Hamaguchi T., Ishida H., Ishiguro M., Ishihara S. (2018). Japanese Society for Cancer of the Colon and Rectum (JSCCR) Guidelines 2016 for the Treatment of Colorectal Cancer. Int. J. Clin. Oncol..

[28] Burton S., Brown G., Daniels I., Norman A., Swift I., Abulafi M., Wotherspoon A., Tait D. (2006). MRI Identified Prognostic Features of Tumors in Distal Sigmoid, Rectosigmoid, and Upper Rectum: Treatment with Radiotherapy and Chemotherapy. Int. J. Radiat. Oncol. Biol. Phys..

[29] Beets-Tan R.G.H. (2013). Pretreatment MRI of Lymph Nodes in Rectal Cancer: An Opinion-Based Review. Colorectal Dis..

[30] Dayal S., Moran B. (2013). LOREC: The English Low Rectal Cancer National Development Programme. Br. J. Hosp. Med..

[31] Arbea L., Ramos L.I., Martínez-Monge R., Moreno M., Aristu J. (2010). Intensity-Modulated Radiation Therapy (IMRT) vs. 3D Conformal Radiotherapy (3DCRT) in Locally Advanced Rectal Cancer (LARC): Dosimetric Comparison and Clinical Implications. Radiat. Oncol..

[32] Mok H., Crane C.H., Palmer M.B., Briere T.M., Beddar S., Delclos M.E., Krishnan S., Das P. (2011). Intensity Modulated Radiation Therapy (IMRT): Differences in Target Volumes and Improvement in Clinically Relevant Doses to Small Bowel in Rectal Carcinoma. Radiat. Oncol..

[33] Engels B., Platteaux N., Van Den Begin R., Gevaert T., Sermeus A., Storme G., Verellen D., De Ridder M. (2014). Preoperative Intensity-Modulated and Image-Guided Radiotherapy with a Simultaneous Integrated Boost in Locally Advanced Rectal Cancer: Report on Late Toxicity and Outcome. Radiother. Oncol..

[34] But-Hadzic J., Velenik V. (2018). Preoperative Intensity-Modulated Chemoradiation Therapy with Simultaneous Integrated Boost in Rectal Cancer: 2-Year Follow-up Results of Phase II Study. Radiol. Oncol..

[35] Li S., Geng J., Wang L., Teng H., Wang Z., Zhu X., Zhang Y., Wang H., Li Y., Cai Y. (2021). Effect of Simultaneous Integrated Boost Intensity Modulated Radiation Therapy (SIB-IMRT) and Non-Operative Strategy on Outcomes of Distal Rectal Cancer Patients with Clinically Positive Lateral Pelvic Lymph Node. Cancer Manag. Res..

[36] Li S., Zhang Y., Yu Y., Zhu X., Geng J., Teng H., Wang Z., Sun T., Wang L., Wang H. (2021). Simultaneous Integrated Boost Intensity-Modulated Radiation Therapy Can Benefit the Locally Advanced Rectal Cancer Patients With Clinically Positive Lateral Pelvic Lymph Node. Front. Oncol..

[37] Bujko K., Nowacki M.P., Nasierowska-Guttmejer A., Michalski W., Bebenek M., Kryj M. (2006). Long-Term Results of a Randomized Trial Comparing Preoperative Short-Course Radiotherapy with Preoperative Conventionally Fractionated Chemoradiation for Rectal Cancer. Br. J. Surg..

[38] Ciseł B., Pietrzak L., Michalski W., Wyrwicz L., Rutkowski A., Kosakowska E., Cencelewicz A., Spałek M., Polkowski W., Jankiewicz M. (2019). Long-Course Preoperative Chemoradiation versus 5 × 5 Gy and Consolidation Chemotherapy for Clinical T4 and Fixed Clinical T3 Rectal Cancer: Long-Term Results of the Randomized Polish II Study. Ann. Oncol..

